# Differential CircRNA Expression Signatures May Serve as Potential Novel Biomarkers in Prostate Cancer

**DOI:** 10.3389/fcell.2021.605686

**Published:** 2021-02-25

**Authors:** John Greene, Anne-Marie Baird, Marvin Lim, Joshua Flynn, Ciara McNevin, Lauren Brady, Orla Sheils, Steven G. Gray, Raymond McDermott, Stephen P. Finn

**Affiliations:** ^1^Department of Histopathology and Morbid Anatomy, School of Medicine, Trinity College, Dublin, Ireland; ^2^Department of Medical Oncology, Tallaght University Hospital, Dublin, Ireland; ^3^School of Medicine, Trinity Translational Medicine Institute, Trinity College, Dublin, Ireland; ^4^Thoracic Oncology Research Group, Trinity Translational Medicine Institute, St. James’s Hospital, Dublin, Ireland; ^5^Department of Medical Oncology, St. Vincent’s University Hospital, Dublin, Ireland; ^6^Department of Histopathology, St. James’s Hospital, Dublin, Ireland

**Keywords:** prostate cancer, circRNA, non-coding RNA, biomarkers, androgen signaling

## Abstract

Circular RNAs (circRNAs), a recently discovered non-coding RNA, have a number of functions including the regulation of miRNA expression. They have been detected in a number of malignancies including prostate cancer (PCa). The differential expression pattern of circRNAs associated with PCa and androgen receptor (AR) status was investigated in this study. circRNA profiling was performed using a high throughout microarray assay on a panel of prostate cell lines, which consisted of normal, benign, and malignant cells (*n* = 9). circRNAs were more commonly significantly up-regulated (*p* < 0.05) than downregulated in malignant cell lines (*n* = 3,409) vs. benign cell lines (*n* = 2,949). In a grouped analysis based on AR status, there were 2,127 down-regulated circRNAs in androgen independent cell lines compared to 2,236 in androgen dependent cell lines, thus identifying a potential circRNA signature reflective of androgen dependency. Through a bioinformatics approach, the parental genes associated with the top 10 differentially expressed circRNAs were identified such as hsa_circ_0064644, whose predicted parental gene target is *RBMS3*, and hsa_circ_0060539, whose predicted gene target is *SDC4*. Furthermore, we identified three circRNAs associated with the parental gene *Caprin1* (hsa_circ_0021652, hsa_circ_0000288, and hsa_circ_0021647). Other studies have shown the importance of *Caprin1* in PCa cell survival and drug resistance. Given the modified circRNA expression signatures identified here, these hypothesis generating results suggest that circRNAs may serve as potential putative diagnostic and predictive markers in PCa. However, further validation studies are required to assess the true potential of these markers in the clinical setting.

## Introduction

Non-coding RNAs (ncRNAs), which include microRNAs (miRNAs) and long non-coding RNAs (lncRNAs) (Shih et al., 2015; Lin et al., 2017; Misawa et al., 2017), play an important role in gene regulation (Warner, 1999; Esteller, 2011; Jeck et al., 2013). circular RNAs (circRNAs), a recently discovered type of ncRNA, are generated from the backsplicing of exons, introns, or both to form exonic or intronic circRNAs (Jeck et al., 2013). circRNAs are covalently joined by their 3′- and 5′- ends, which are formed by back-splice events, thus presenting as closed continuous structures, making them highly stable and resistant to degradation (Salzman et al., 2012; Barrett et al., 2015). circRNAs have many postulated functions such as the regulation of miRNA function by controlling the expression of miRNAs through a “sponging effect,” however this appears to apply to only a small number of circRNAs, such as ciRS-7 acting as a miR-7 sponge (Bahn et al., 2015). It has also been proposed that circRNAs may have a role in protein synthesis, with a number of circRNAs implicated in the translation of peptides, such as ZNF609 (Legnini et al., 2017). As research in this space increases, it is now clear that the circRNA-miRNA-mRNA network, plays an important role in both gene regulation and carcinogenesis (Su et al., 2019). circRNAs have also been detected in prostate cancer, which make them an attractive research target (Greene et al., 2019). PCa growth and development is primarily dependent on the androgen receptor (AR), which is the target of therapeutic agents such as enzalutamide and abiraterone (Antonarakis et al., 2014). AR copy number gain and generation of variants such as AR-V7 are associated with the development of castration-resistant disease and drug resistance, however, despite significant advances; no suitable assay has become routinely available in the clinic to identify resistance mediators of AR targeting therapeutics (Antonarakis et al., 2014; Romanel et al., 2015). Currently, PSA is used to screen men for PCa, yet when used in isolation, it is sub-optimal given issues with specificity and sensitivity (O’Sullivan, 2017). Similarly, PSA cannot reliably detect early relapse or resistance to drug treatments, nonetheless earlier detection of castration-resistant disease would allow for more appropriate management of these patients and therefore significantly improve outcomes (Di Nunno et al., 2018). Thus, there is a need to identify new diagnostic and predictive biomarkers, as well as new therapeutic agents for use in the clinical setting. circRNAs may fulfill these roles, given their expression in PCa, in addition to studies showing circRNA such as circFOXO3 (hsa_circ_0006404) acting as an miRNA sponge in this disease (Kong et al., 2020).

The aim of this study was to profile circRNAs in a panel of prostate cell lines and identify potential signatures associated with malignancy and androgen dependency. We propose that circRNAs have the potential to serve as useful biomarkers to improve diagnostic screening for PCa and/or identify men who are at risk of developing castration-resistant disease.

## Materials and Methods

### Cell Lines

A panel of malignant and benign prostate cell lines were used in this project, all of which were purchased from the ATCC (LGC standards, Middlesex, United Kingdom). The benign prostate cell lines (PWR-1E, RWPE-1) were cultured in Keratinocyte Serum Free Media (Thermo Fisher Scientific, CA, US), containing 0.05 mg/mL bovine pituitary extract (BPE) and 5 ng/mL Epidermal growth factor (EGF) (Thermo Fisher Scientific) and 1% Penicillin Streptomycin (P/S) (Merck KGaA, Darmstadt, Germany). The benign prostatic hyperplasia (BPH-1) and PCa cell lines (22Rv1, VCaP, LNCaP, DU145) were cultured in RPMI-1640 (Merck KGaA) supplemented with 10% Fetal Bovine Serum (FBS) (Merck KGaA) and 1% P/S. PC-3 was cultured in ATCC-formulated F-12K Medium (Thermo Fisher Scientific) supplemented with 10% FBS and 1% P/S.

### RNA Preparation

Total RNA was prepared from cell lines (biological triplicates) according to manufacturer’s instructions. Briefly, cells were lysed directly with 2 mL TRIzol^®^ Reagent (Thermo Fisher Scientific). For phase separation, 200 μL chloroform (Merck KGaA) was added to the cell lysate. The sample were vortexed vigorously for 15 s, incubated at RT for 5 min and centrifuged at 12,000 × g for 15 min at 4°C. The upper aqueous phase was transferred into a fresh tube and the RNA precipitated from the aqueous phase, by the addition of 400 μL isopropyl alcohol (Merck KGaA). The sample was mixed, incubated for 5 min at RT and centrifuged at 12,000 × g for 15 min at 4°C. One milliliter 75% EtOH (Merck KGaA) was added to pellet, and centrifuged at 12,000 × g for 10 min at 4°C. The EtOH was removed and a repeat pulse centrifuge was performed. Surplus EtOH was removed and the pellet air-dried. The sample was re-suspended in 30 μL molecular grade H_2_O before DNase treatment with Ambion^®^ TURBO^TM^ DNase (Thermo Fisher Scientific), and an additional RNA clean-up using standard EtOH precipitation.

### CircRNA Microarray

The panel of cell lines (three biological replicates for each) were profiled using the Arraystar Human circRNA Array version 2.0 (Arraystar, MD, United States). The sample preparation and microarray hybridization were performed according to manufacturer’s instructions. Briefly, total RNA was digested with RNAse R (Epicenter, Illumina, CA, United States) to remove linear RNAs and enrich for circRNAs. The enriched circRNAs were amplified and transcribed into fluorescent cRNA using a random priming method with Arraystar Super RNA Labeling Kit (Arraystar). The labeled cRNAs were hybridized onto the Arraystar Human circRNA Array V2 (8 × 15 K). The array slides were washed and scanned on the Agilent Scanner G2505C. Agilent Feature Extraction software (version 11.0.1.1) was used to analyze acquired array images.

### Microarray Data Analysis

Quantile normalization and subsequent data processing were executed using the R software package (R Core Team, 2020). circRNAs with at least 4 out of 8 samples that were flagged as present or marginal (an attribute that denotes the quality of the entities) were considered as target circRNAs according to GeneSpring software’s definitions and instructions. circRNA and miRNA interactions were predicted with the Arraystar’s miRNA target prediction software based on TargetScan (Agarwal et al., 2015) and miRanda (John et al., 2004). These target circRNAs were used for further differential analysis. A student’s paired *t*-test was used to identify significantly altered circRNAs. The false discovery rate (FDR) was applied to determine the threshold of *p*-value. An FDR of < 0.05 was used. Changes in expression were identified using unsupervised clustering analysis (euclidean distance measure and the “average” agglomeration method) and associated heat-maps generated.

## Results

### CircRNAs Are Differentially Expressed in Malignancy

The human circular RNA microarray (Arraystar) version 2.0 covers 13,617 previously discovered human circRNAs. Our cell line panel consisted of malignant (22Rv1, LNCaP, VCAP, DU145, and PC-3), benign (BPH-1), and normal (PWR1, RWPE-1) cell lines. AR expression in 22Rv1, LNCaP, and VCaP was confirmed using qPCR (Supplementary Methods and Supplementary Figure S1). In total 9,805 circRNAs were classified as present across the complete panel of cell lines. Initially, circRNA expression levels between cell lines based on malignancy was investigated. Differentially expressed circRNAs were examined by computing the fold change (FC) (i.e., the ratio of the group averages) for each circRNA between malignant, normal and benign cell lines. Associated parental genes for the top 10 altered circRNAs were predicted from circBase, which uses the co-ordinates from each detected circRNA to identify the associated gene from the UCSC genome database (Glazar et al., 2014).

**FIGURE 1:**
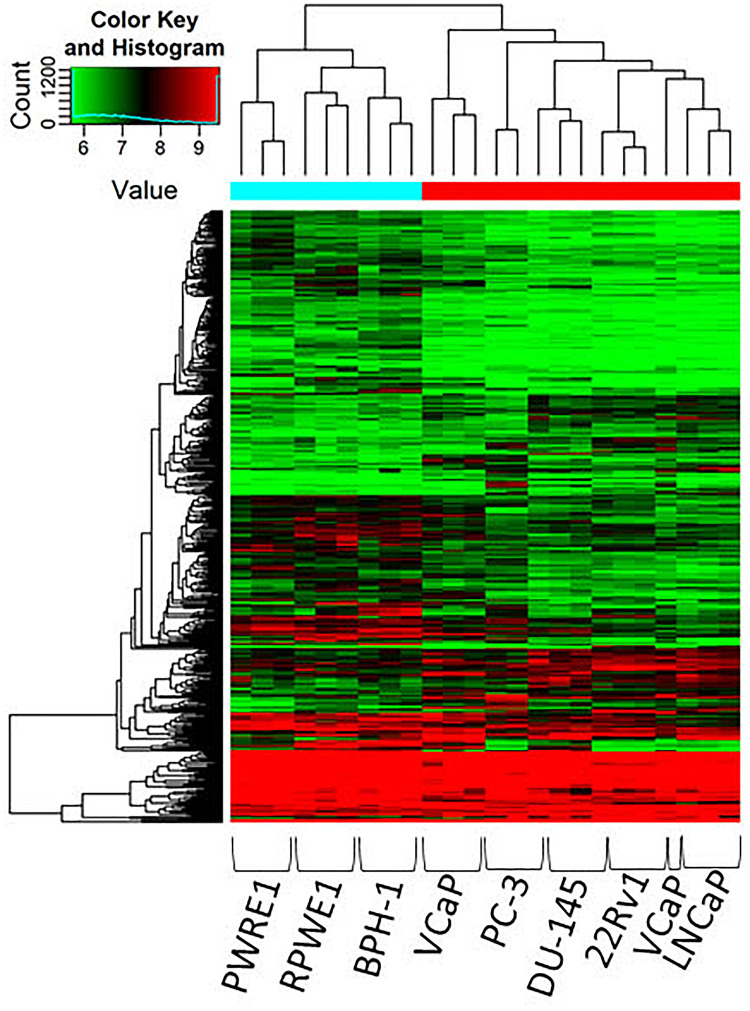
Clustering heatmap of microarray data showing differential expression of circRNAs between malignant, benign and normal cell lines. Unsupervised clustering (euclidean distance measure and the “average” agglomeration method) was used for analysis (*n* = 3). circRNAs were more likely to be down-regulated in normal and benign cell lines compared to malignant cells.

CircRNAs were significantly differentially expressed between these groups (BPH vs. normal; malignant vs. BPH/normal) according to FC and *p*-value (Figure 1). We first examined the difference in expression of circRNAs between normal cell lines (PWR1, RWPE-1) and the benign cell line, BPH-1. CircRNAs were more often down-regulated in normal cell lines compared to the benign cell line (5,429 vs. 4,344). Next, we examined the expression of circRNAs between the normal/benign cells compared to malignant cells. In total, a higher number of circRNAs were down-regulated in normal/benign cells compared to malignant cells (5,300 vs. 4,457). The top 10 down and up-regulated circRNAs according to FC, along with their associated parental gene, are given in Tables 1, 2, respectively.

**TABLE 1:** Top 10 down-regulated circRNAs in PCa (malignant vs. normal/benign cell lines)*.

circRNA	FC	Chromosome	Parental gene
hsa_circ_0064644	5.1	chr3	*RBMS3* (Zhu et al., 2019)
hsa_circ_0082672	5.1	chr7	*HIPK2* (Imberg-Kazdan et al., 2013)
hsa_circ_0022382	3.3	chr11	*FADS2*
hsa_circ_0049657	3.0	chr19	*NFIX* (Grabowska et al., 2014)
hsa_circ_0005402	3.0	chr15	*ANXA2* (Griner et al., 2015)
hsa_circ_0082680	2.9	chr7	*HIPK2* (Imberg-Kazdan et al., 2013)
hsa_circ_0001187	2.5	chr21	*DOPEY2*
hsa_circ_0082546	2.5	chr7	*MTPN*
hsa_circ_0022383	2.5	chr11	*FADS2*
hsa_circ_0056731	2.4	chr2	*CACNB4*

*References refer to the previous identification of these genes in cancer.

**TABLE 2:** Top 10 up-regulated circRNA in malignant vs. benign/normal cell lines*.

circRNA	FC	Chromosome	Parental gene
hsa_circ_0060539	7.8	chr20	*SDC4* (Leblanc et al., 2018)
hsa_circ_0001589	6.3	chr6	*HIST1H1D*
hsa_circ_0026457	6.0	chr12	*KRT5* (Du et al., 2019)
hsa_circ_0043570	5.0	chr17	*TNS4* (Muharram et al., 2014)
hsa_circ_0060540	5.0	chr20	*SDC4* (Leblanc et al., 2018)
hsa_circ_0008805	4.8	chr17	*ARHGAP23*
hsa_circ_0084021	3.4	chr8	*PLEKHA2* (Bii et al., 2018)
hsa_circ_0026358	3.2	chr12	*KRT7* (Thomas et al., 2016)
hsa_circ_0007058	3.2	chr11	*TEAD1* (Knight et al., 2008)
hsa_circ_0004405	2.8	chr5	*FAM169A*

*References refer to the previous identification of these genes in cancer.

A number of circRNAs, that could play a role in PCa, were identified through their interaction with their associated parental genes. hsa_circ_0064644 was the highest down-regulated circRNA (FC 5, *p* = 0.001), located on chromosome 3 (Table 1). Its’ predicted parental gene target using circBase is *RBMS3*, which has previously been identified to suppress cell proliferation, migration, invasion, and angiogenesis in a number of different cancers (Zhu et al., 2019). *RBMS3* has also been shown to act as a miRNA sponge in PCa via lncRNAs. Other genes of interest include *HIPK2* (hsa_circ_0082672) and *NFIX* (hsa_circ_0049657), both known to be involved in the regulation of the AR (Imberg-Kazdan et al., 2013). hsa_circ_0005402, is linked to *ANXA2*, which has been suggested to play a role in epithelial-mesenchymal transition (EMT) in PCa (Yang et al., 2018) via transcriptional repression by ERG (Griner et al., 2015). hsa_circ_0060539, located on chromosome 20, was the highest up-regulated circRNA. The predicted gene target for this circRNA is *SDC4*, a known active gene in PCa (Griner et al., 2015). Similarly, *KRT5* (hsa_circ_0026457) has been shown to be involved in the inhibition of PCa stem cell invasion, migration and proliferation, as has *KRT7* (hsa_circ_0026358), which has been identified along with *FAM129A*, *MME* and *SOD2* as part of a four-gene androgen regulated panel (Thomas et al., 2016).

### CircRNAs Are Differentially Expressed According to Androgen Dependence

**FIGURE 2:**
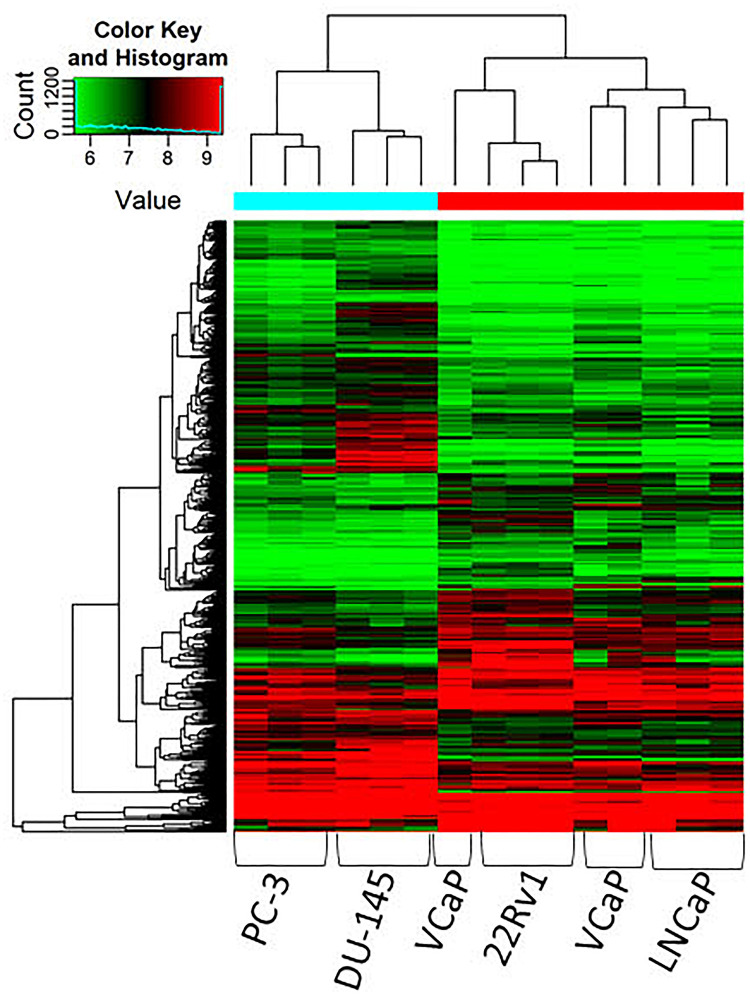
Clustering heatmap showing differential expression of circRNAs between AR dependent cells LNCaP, 22Rv1 and VCaP (hormone sensitive) and AR independent cells DU145 and PC-3 (castration resistant). Unsupervised clustering (euclidean distance measure and the “average” agglomeration method) was used for analysis (*n* = 3).

Lastly, the differing expression of circRNAs in relation to androgen sensitivity was examined, which we hypothesized would reflect the development of castration resistant PCa. Cell lines were stratified into two groups based on AR status: androgen dependent/castration-sensitive (22Rv1, LNCaP, VCaP) and androgen independent/castration resistant) cell lines (DU145, PC-3). Response to androgen ablation was demonstrated by confirming the presence or absence of AR-FL across cell lines (see Supplementary Figure S1). CircRNAs were more often down-regulated in cell lines that were androgen independent compared to androgen-dependent cell lines (5,067 down-regulated vs. 4,693 up-regulated, respectively) (Figure 2). The top 10 down and up-regulated circRNAs and their associated parental genes are given in Tables 3, 4, respectively. The up-regulated hsa_circ_0000361 (FC 19.2, *p* < 0.001) is an exonic circRNA located on chromosome 11 and is associated with the gene *SIAE* (Table 3). mRNA expression of *SIAE* has previously been shown to be reduced in patients with cancer (Oh et al., 2020). We identified three circRNAs associated with the parental gene *Caprin1* (hsa_circ_0021652, hsa_circ_0000288, and hsa_circ_0021647). *Caprin1* plays an important role in PCa cell survival and its upregulation has been shown to be associated with drug resistance, and is increased in SPOP mutated cell lines (Shi et al., 2019).

**TABLE 3:** Top 10 up-regulated circRNAs in androgen dependent vs. independent cell lines*.

circRNA	FC	Chromosome	Parental gene
hsa_circ_0000367	19.2	chr11	*SIAE* (Oh et al., 2020)
hsa_circRNA_404686	18.5	chr1	*GPR137B* (Du et al., 2015)
hsa_circ_0004183	17.7	chr10	*FRMD4A* (Zheng et al., 2016)
hsa_circ_0001666	13.6	chr6	*FAM120B*
hsa_circRNA_400850	13.3	chr11	*TRAPPC4*
hsa_circ_0021652	13.0	chr11	*CAPRIN1* (Shi et al., 2019)
hsa_circ_0000288	12.7	chr11	*CAPRIN1* (Shi et al., 2019)
hsa_circ_0021647	12.4	chr11	*CAPRIN1* (Shi et al., 2019)
hsa_circ_0006220	11.4	chr17	*TADA2A*
hsa_circ_0043278	11.4	chr17	*TADA2A*

*References refer to the previous identification of these genes in cancer.

**TABLE 4:** Top 10 down-regulated circRNAs in androgen dependent vs. independent cell lines*.

circRNA	FC	Chromosome	Parental gene
hsa_circ_0000825	40.6	chr18	*MTCL1*
hsa_circ_0084615	24.1	chr8	*ASPH* (Barboro et al., 2020)
hsa_circ_0002083	18.2	chr11	*ETS1* (Rodgers et al., 2019)
hsa_circ_0091934	11.7	chrX	*FLNA* (Ravipaty et al., 2017)
hsa_circ_0091894	10.3	chrX	*FLNA* (Ravipaty et al., 2017)
hsa_circ_0085923	9.1	chr8	*PLEC* (Martiny et al., 2018)
hsa_circ_0007386	8.8	chr2	*CRIM1* (Hudson et al., 2015)
hsa_circ_0044468	8.6	chr17	*ITGA3* (Kurozumi et al., 2016)
hsa_circRNA_402986	7.9	chr3	*PLOD2* (He et al., 2018)
hsa_circ_0025506	7.5	chr12	*GPRC5A* (Sawada et al., 2020)

*References refer to the previous identification of these genes in cancer.

The highest down-regulated circRNA by FC was hsa_circ_0000825 (FC 40, *p* < 0.0001) located on chromosome 18 and is associated with the gene *MTCL1* (Table 4). This gene has previously been identified to play a role in the development of castration-resistant PCa (Zhang et al., 2018). Other parental genes of interest include *FLNA* (hsa_circ_0091934 and hsa_circ_0091894) located on the X chromosome, which plays a significant role in PCa development and progression, and has been highlighted as a possible biomarker for disease screening and detection (Ravipaty et al., 2017).

## Discussion

CircRNAs are a novel type of ncRNA which have been identified across a range of cancers including PCa (Lim et al., 2018; Greene et al., 2019). The most abundant class of ncRNA are lncRNAs which have been extensively investigated in PCa (Ramnarine et al., 2019). A number of lncRNAs have been identified as potential predictive and prognostic biomarkers including SChLAP1, UCA1 and PCAT14 (Mehra et al., 2016; Fotouhi Ghiam et al., 2017; White et al., 2017). Further work is exploring lncRNAs as therapeutic targets in PCa using small interfering RNAs (siRNAs), antisense oligonucleotides (ASOs) and small molecule inhibitors (Fu et al., 2020). Therefore, there is significant interest in the role ncRNAs may play in the future management of PCa. The exact role of circRNAs in cancer has yet to be fully elucidated, however initial studies suggest circRNAs may play a role in cancer development, progression and the development of resistance to therapeutic agents (Greene et al., 2017). CircRNAs have been identified as potential biomarkers in cancer due to their abundance, stability and disease specific activity (Dong et al., 2017). The identification of circRNAs has been complicated by bioinformatics challenges, however the development of improved detection methodologies and statistical methods has aided circRNA research, particularly with improvements in reducing false-positive detection rates (Szabo and Salzman, 2016). In PCa, a number of circRNAs have been identified which have been identified as potential biomarkers (Tucker et al., 2020). Our laboratory previously identified the circRNA, hsa_circ_0004870, to be associated with resistance to the androgen receptor inhibitor enzalutamide (Greene et al., 2019). Other circRNAs of note that have been identified in PCa include circAMACR, circAURKA and circAR3 which is encoded by the AR gene (Chen et al., 2019).

We identified a number of circRNAs that could represent a unique gene signature of aggressive PCa. Initial bioinformatics analysis identified a significant number of differentially expressed circRNAs between malignant vs. benign/normal cell lines. There was a trend for circRNAs to be down-regulated in normal compared to benign cells. This trend continued when we compared normal/benign cells to malignant cells. When we examined circRNA expression in cell lines according to androgen dependency, we found circRNAs again to be more often down-regulated in androgen independent cell lines, reflective of aggressive PCa.

Parental genes of interest were *RBMS3* (hsa_circ_0064644) and *SDC4* (hsa_circ_0060539). Interestingly, we identified three circRNAs associated with the parental gene *Caprin1* (hsa_circ_0021652, hsa_circ_0000288, and hsa_circ_0021647) and two circRNAs associated with *FLNA* (hsa_circ_0091934 and hsa_circ_0091894). Many of these genes are currently under active investigation as biomarkers in PCa, and identifying their associated circRNAs may contribute to ongoing research in this field (Ravipaty et al., 2017; Shi et al., 2019; Zhu et al., 2019; Jiang et al., 2020).

There is evidence to suggest that circRNAs regulate miRNA function by competing for the pool of miRNA binding sites to influence the activities of miRNAs in regulating gene expression (Hansen et al., 2013). Interestingly, a number of miRNAs that have been detected in PCa are linked to the AR pathway (Budd et al., 2015; Fabris et al., 2016). miRNA binding sites can be predicted using a bioinformatics pipeline for each detected circRNA that identified an associated gene. For instance, for hsa_circ_0064644, the predicted miRNA is miR-15a, which has been show to play a role in PCa progression and metastases (Jin et al., 2018). Therefore, predicted miRNAs could be used to identify circRNAs that play a role in gene regulation in PCa, and further our understanding of the importance of the circRNA-miRNA-mRNA network in this disease.

This study had several limitations. Firstly, we used a circRNA microarray platform to detect circRNAs compared to RNA-seq methods which may have higher sensitivity and accuracy to detect circRNAs. Secondly, this was a preliminary *in vitro* study which identified multiple potential genes and ongoing validation is required in cell lines and *in vivo* to confirm our findings here.

## Conclusion

In conclusion, circRNAs were modified between benign and malignant cell lines, as well as androgen dependent and independent cell lines, which suggests a role for circRNAs in PCa initiation and progression. This study highlights the importance of circRNAs in PCa and their potential role in identifying the disease and monitoring response to treatment. The dysregulated signature identified here may prove useful for the development of a blood-based assay with both diagnostic and predictive value in this disease. However, this would require validation in larger patient cohorts.

## Data Availability Statement

The raw data supporting the conclusions of this article will be made available by the authors, without undue reservation.

## Author Contributions

All authors listed have made a substantial, direct and intellectual contribution to the work, and approved it for publication.

## Conflict of Interest

The authors declare that the research was conducted in the absence of any commercial or financial relationships that could be construed as a potential conflict of interest.
