# Supplementary material for: Differential CircRNA Expression Signatures May Serve as Potential Novel Biomarkers in Prostate Cancer
Source: Front Cell Dev Biol. 2021 Feb 25;9:605686. doi: 10.3389/fcell.2021.605686 (PMC7946979; doi:10.3389/fcell.2021.605686)
Supplement: Supplementary file 1 [file Table_1.DOCX]

**Supplementary data**

1. **Methods**
2. **Figure S1**

**Quantitative real-time PCR**

cDNA was synthesized from 1 µg RNA using a High Capacity cDNA Reverse Transcription Kit (Thermo Fisher Scientific) according to manufacturer’s instructions. qPCR was performed on a 7500 Real-Time PCR System using SYBR™ Green (Thermo Fisher Scientific). GAPDH was used as a reference gene. The relative expression and fold change of each gene was calculated using the delta delta Ct method.

**Standard curve method**

Standard curves for known gene copy numbers using gBlocks® gene fragments (Integrated DNA Technologies, CA, US) were designed for AR-FL (Forward: 5'CAGCCTATTGCGAGAGAGCTG-3', Reverse: 5'-GAAAGGATCTTGGGCACTTGC-3'). A standard curve was prepared using serial dilution so that AR-FL was present at 300,000 copies, 30,000 copies, 3,000 copies, 300 copies and 30 copies. Threshold cycle numbers from qPCR reactions were determined for cDNA specific to gene copy number at 6 dilutions containing the indicated number of copies of each transcript. Formulas were derived to quantify the absolute copy numbers on the basis of Ct values.

**Androgen Receptor (AR) expression in cell lines**

AR expression was confirmed by qPCR, with AR-FL detected in all known AR dependent cell lines (22Rv1, LNCaP, VCaP) compared to AR independent cell lines (DU145, PC-3 and BPH-1) (Fig. S1). VCaP cell lines had the highest copy number of AR-FL (5.75 ± 0.03) compared to LNCaP (5.04 ± 0.19) (p≤0.05), and 22Rv1 (4.55 ± 0.19) (p≤0.01).

**Figure S1**. AR-FL was expressed in all 22Rv1, LNCaP and VCaP.

AR-FL was not detected in known AR independent lines, DU145, PC-3 and BPH-1 cell lines (Data not shown). Data graphed as mean ± SEM (n=3). Statistical analysis performed using ordinary one-way ANOVA (* p≤0.05, ** p≤0.01).
